# The Royal Waterloo Hospital for Children and Women

**Published:** 1904-01-23

**Authors:** 


### THE ROYAL WATERLOO HOSPITAL FOR CHILDREN AND WOMEN.

The eighty-eighth annual Court of Governors of this hospital was held at the Mansion House on Monday last, the Lord Mayor being in the chair. The Lady Mayoress was also present together with Mrs. Henry Tate, Sir E. Durning-Lawrence, Mr. J. Topham Richardson and many others.

The Lord Mayor moved the adoption of the report for 1903, and the re-election of the treasurer, chairman, and other officers of the institution and that the annual Court of Governors should record its best thanks to them for the work which had been so ably carried out during the past year, and express entire satisfaction at the results of their efforts. The Chairman went on to say that this hospital was the oldest of its kind in London. having been established in 1816. Until quite recently it had only contained sufficient accommodation for 50 beds and its settled income was not more than £705, of which £300 only was completely secured. The sum now required to equip the hospital with necessary buildings, new furniture, surgical appliances, etc., was £60,000; a comparatively large sum though small when compared to the demands put forth by other hospitals for £500,000, £300,000, etc., but sufficiently embarrassing to the Governors of the hospital to have to collect. If this institution was to be carried on in an efficient manner, it was absolutely necessary that some effort should be made in order to obtain the required sum. The hospital had formerly been carried on at an annual cost of about £3,000, but the up-keep of the new hospital would probably amount to about £15,000 annually or about £12,000 more than was required before. This matter was greatly disturbing the minds of the Governors, for they were extremely anxious that the hospital should be carried on in a manner suitable to the requirements of the patients, and they looked to their friends and to the public for the support that the institution deserved. The hospital was placed in a somewhat out-of-the way spot, not much frequented by the wealthy, who, therefore, had not the opportunity of seeing the great and good work that was being carried on there. The hospital rendered assistance to women in their hour of need and to children. He strongly appealed for the money which was so badly wanted by the hospital, although, unfortunately, times were bad at present, and people had less to give away. He was of opinion that hospitals were used more than they ought to be by persons who could well afford to pay. Having paid a tribute to the excellent services rendered by the chairman of the hospital, Sir E. Durning-Lawrence, the Lord Mayor went on to say that any money given to that hospital was well dispensed, as the work was carefully conducted and thoroughly supervised, and the institution was one that deserved warm support.

Dr. Septimus Sunderland, in seconding the resolution, said that speaking from a knowledge of the hospital resulting from weekly visits during 15 or 16 years, he wished to state that the class of patients was the poorest of the poor, and there was no fear of the money contributed by the subscribers going into improper channels.

Mr. J. Topham Richardson, the treasurer of the hospital, moved a resolution to the effect that the hospital was in every way worthy of support and had strong and peculiar claims upon the City of London; also that a subscription list should at once be opened to make up the annual deficit of £2,000, which would be materially increased when the new hospital was opened, and that donations should be invited towards the sum required for the rebuilding and extension which was then in progress. The treasurer referred to the strong and peculiar claims which the hospital had on the City of London on account of its being the oldest of its kind, and secondly because it had been distinctly proved some years back to be wholly inadequate to meet the needs of the patients. They had found themselves compelled during the past year to have recourse to advertisement, which had consisted chiefly in an illuminated captive balloon; this measure had been necessary in order to arouse interest in their work, which had been carried on quietly for so long in a very poor neighbourhood. Having paid a tribute to the services rendered by the matron and to the harmony that existed between the medical staff and the sisters and nurses he ended by saying that they had every reason to believe that during the next 18 months they would be proud to have contributed to the erection of a building that would be a credit to the City of London.

The resolution having been seconded by Mr. E. M. Shemeld a vote of thanks to the honorary medical staff was proposed by Mr. J. Watson-Smyth, seconded by Mr. J. Topham Richardson, and replied to by Dr. Septimus Sunderland.

Mr. Seymour Eastwood then proposed a vote of thanks to the Duchess of Albany and the Ladies' Committee for the great work they had undertaken on behalf of the hospital, and said that the hospital had always been much indebted to ladies both in the way of paying visits to it and sending presents. Having eulogised the energy and devotion shown by the Duchess of Albany in her endeavours to help forward the work of the hospital, he pointed out that they were also indebted to the Countess of Derby who had placed Derby House at the disposal of the Ladies' Committee for the purpose of holding their meetings there. He then briefly alluded to the forthcoming ball at Covent Garden on February 2nd under the auspices of the ladies' committee. The vote of thanks was seconded by Mr. F. C. Eden.

Sir E. Durning-Lawrence then moved a resolution enabling the Board of Governors to alter any of the rules of the hospital, and explained that this step was found necessary in view of the reconstruction and enlargement of the hospital. Up to the present they had been unable to make any alteration in the rules except at the annual Court of Governors. The resolution was seconded by Mr. W. Symes, and Sir E. Durning-Lawrence proposed a vote of thanks to the Lord Mayor for presiding, and to the Lady Mayoress for being present; this was seconded by the Rev. Dr. Walpole, rector of Lambeth.

The Lord Mayor, in replying, said that if he had the wealth of Mr. Carnegie, instead of giving libraries he would bestow the greater part of it on the hospitals which were doing such a great and beneficent work.

